# Examining the test–retest reliability of commonly used neuromuscular, morphological, and functional measures in aging adults

**DOI:** 10.1007/s11357-025-01590-0

**Published:** 2025-03-11

**Authors:** Gustavo Z. Schaun, Peter Raidl, Luana S. Andrade, Gabriela B. David, Eduardo F. Marins, Mariana S. Häfele, Stephanie S. Pinto, Robert Csapo, Cristine L. Alberton

**Affiliations:** 1 Centre for Sport Science and University Sports, Department of Sport and Human Movement Science, University of Vienna, Auf Der Schmelz 6, 1150 Vienna, Austria; 2 Neuromuscular Assessment Laboratory, Physical Education School, Federal University of Pelotas, Pelotas, RS Brazil

**Keywords:** Physical function, Functional performance, Measurement precision, Reproducibility, Agreement

## Abstract

The present study aimed at determining the test–retest reliability of commonly used measures in a sample of middle-aged and older participants who presented with a wide range of functional abilities. Participants were middle-aged (40–55 years) and older adults (> 60 years) with- and without mobility limitations, tested both before and after a 4-week period (n = 43). Measures included maximal dynamic (1-RM) and isometric strength, surface EMG (sEMG), peak power, ultrasound-derived muscle size and quality, as well as the performance on the 30-s sit-to-stand, habitual and maximal gait speed, timed up-and-go, stair climb, and 6-min walk tests. Reliability was assessed by means of the intraclass correlation coefficient (ICC), the standard error of measurement (SEM), the minimal detectable change (MDC), and the coefficient of variation (CV). Both dynamic and isometric strength measures displayed excellent reliability (ICCs = 0.96–0.99; CV = 2.2%-7%), whereas muscle size and quality (ICCs = 0.88–0.98; CV = 3.3%-8.7%), functional performance (ICCs = 0.78–0.92; CV = 4.2%-6.8%) and peak power (ICCs = 0.76–0.98; CV = 6.6%-12.8%) results showed good to excellent reliability. Peak power at 80–90% 1‑RM and sEMG measures, on the other hand, showed larger absolute error (CV = 14.4%-18.3% and CV = 14.3%-19.8%, respectively), despite good relative reliability (ICCs = 0.85–0.86). Further results include the comparison between the three subsets of participants included. Our main analysis suggests that most of these measures are sufficiently reliable, even when the two tests are performed a month apart from each other. Our study also supports the notion that, provided that the same equipment and procedures are used, the test–retest reliability of the measures is mostly comparable between the subsets of participants investigated.

**Supplementary Information:**

The online version contains supplementary material available at 10.1007/s11357-025-01590-0.

## Introduction

Aging is typically associated with several physiological and functional impairments, such as reductions in muscle strength and power [1–5]. These impairments, in turn, increase the risk of falls, loss of independence, disability, and mortality in older individuals [6–12]. Thus, there is great interest in identifying interventions that can prevent or at least mitigate some of these age-related impairments.

Resistance exercise is currently recommended as a safe and effective approach to combat these impairments and their debilitating consequences in both middle-aged and older adults [13–15]. In fact, even in the presence of age-related diseases, increasing muscle mass, strength, and power holds great potential for reducing the incidence of disability in these individuals [7]. When evaluating the effectiveness of the respective training intervention, it is imperative that the outcome measures used are reliable, demonstrating consistency over time.

Previous research has shown several measures of physical and functional capacity to be reliable when assessed in different cohorts of older individuals (e.g., [16–21]), but two aspects become evident when considering the literature. First, most studies typically investigate the test–retest reliability of outcomes within a narrow timeframe (e.g., ≤ 1 week), whereas most training studies and clinical trials employ several weeks of intervention before participants are retested. If the intervention applies a similar stimulus to that of the test (e.g., using the same exercise for training and one-repetition maximum (1‑RM) test), this may not pose a problem. However, it is not uncommon for several other tests to be applied alongside the practiced one.

In addition, it is often the intention of researchers to compare different groups of individuals, such as young and older adults or healthy individuals and patients suffering from chronic disease. Reliability studies, however, often investigate the reliability of the measures in only one of these groups, and very little is known about the differences in reliability between such groups when the same procedures, equipment, and environment are employed.

Therefore, the aim of the present study was to determine the test–retest reliability of several different neuromuscular, morphological, and functional tests in a sample of middle-aged and older adults. It further sought to compare the test–retest reliability of these measures between three subsets of participants, namely middle-aged adults, healthy older adults, and older adults with mobility limitations who were all tested using the same procedures and equipment.

## Materials & methods

This study leverages data from a larger trial that aimed to compare the neuromuscular, morphological, and functional adaptations of the three aforementioned groups in response to a 12-week high-velocity resistance training program (HVRT; [22]). Specifically, data from before and after a 4-week control period were analyzed in the current investigation and used to determine the test–retest reliability of the outcomes of interest, all of which are novel and not yet published.

Participants were recruited from the urban area of Pelotas, Brazil, through advertisements published in newspapers and social media and included middle-aged (40–55 years) and older adults (> 60 years) with- and without mobility limitations, classified on the basis of a Short Physical Performance Battery score ≤ 9 (mobility-limited) or ≥ 10 (healthy). For more information on the recruitment and enrollment details, readers are referred to the main trial paper by Schaun et al. [22]. After giving their written informed consent (CAAE nº 13459719.0.0000.5313), participants were familiarized with the study procedures in two separate sessions. Participants then attended two testing sessions in which all the outcomes of interest were measured. On the first day of testing, ultrasound images were taken from the knee extensor muscles, followed by a maximal knee extension (KE) voluntary isometric contraction (MVIC) test coupled with surface electromyography (sEMG) measures. MVIC testing was followed by a KE one-repetition maximum (1-RM) and a peak power test, 20–30 min apart from the KE 1-RM test. Separated by at least 72 h, the second day of testing included a leg press (LP) 1-RM test, also followed by an LP power test, and a battery of different functional tests. The tests performed on both the first and second day of testing were repeated after a 4-week control period by the same investigators, in the same order, following the same procedures, and at the same time of day (± 2 h).

### Measures

#### Ultrasound testing

After a 5-min resting period in the supine position, five ultrasound images were taken from the rectus femoris (RF), vastus intermedius (VI), vastus lateralis (VL) and vastus medialis (VM) muscles using a B-mode ultrasound device (Tosbee/SSA-240a, Toshiba®, Japan) and a 7.5 MHz linear-array probe positioned perpendicularly to the muscle of interest (for a detailed explanation of probe positioning, the reader is referred to [22]). Muscle thickness (MT) and echo intensity (EI) measures were determined for each muscle and are presented as the average of the five images per muscle. In addition, an overall quadriceps muscle thickness and echo intensity measure were also determined as the sum and the average of the individual muscle values, respectively. Specifically, RF, VL, and VM muscle thicknesses were defined as the distance between the superficial and deep muscle aponeurosis, whereas VI thickness corresponded to the distance between the superficial aponeurosis and the hyperechoic interface of the femur. Muscle quality was determined based on the muscles’ EI values using a grayscale analysis function on the ImageJ software, and expressed in arbitrary units between 0 (black, higher muscle quality) and 255 (white, lower muscle quality).

#### Maximal dynamic strength

Maximal dynamic strength was assessed using the KE and LP 1-RM tests on separate days. Both tests were preceded by a 10-repetition warm-up set at 50% of the estimated 1-RM load, determined during the familiarization, and each participant had up to 5 attempts per exercise, with 3 min between attempts. Resistance was readjusted based on the Lombardi scale and the 1-RM load (in kg) was considered as the resistance at which participants could only perform one repetition with both proper technique and range of motion. Range of motion was registered at baseline using a custom-built device [23] and re-assessed at the pre-intervention time point.

#### Peak power

After completing each of the 1-RM tests, participants rested for 20–30 min and performed a peak power test for the respective exercises (i.e., KE or LP). Specifically, each participant performed one repetition of the exercise at loads corresponding to 30%, 40%, 50%, 60%, 70%, 80%, and 90% of 1‑RM, with the repetitions separated by 1-min intervals. For this purpose, they were requested to perform the concentric portion of the movements as fast as possible and the eccentric phase in 2 s. During the concentric portion of each repetition, peak power was determined using a linear position transducer (Chronojump, BoscoSystem®, Barcelona, Spain) attached to the weight stack on the KE machine and perpendicular to the motion trajectory on the LP machine sled to ensure a direct vertical displacement in both exercises. For logistic reasons, the order of the loads was not randomized, but it was kept constant at both baseline and pre-intervention.

#### Maximal voluntary isometric contraction

Individuals were positioned on a KE machine (NEWFIT®, Cascavel, Brazil) at 90º of hip and knee flexion and instructed to exert maximal force as fast as possible while receiving strong verbal encouragement. Three ~ 5 s attempts were performed, with 2 min recovery between them, and maximal isometric force was measured using a load cell (Miotec®, Porto Alegre, Brazil). During the MVIC test, the neuromuscular activation of the RF and VL muscles were also measured using sEMG (Miotool400, Miotec®, Porto Alegre, Brazil). Electrode positioning followed established guidelines [24] and a detailed explanation is available elsewhere [22]. To ensure consistent positioning between baseline and pre-intervention time points, electrode positions were carefully mapped on semi-transparent polypropylene sheets in the first session and replicated when the test was repeated at the end of the control period [25]. Force signals were filtered using a low-pass fifth-order Butterworth digital filter at a cutoff frequency of 8 Hz and sEMG signals were band-pass filtered by a fifth-order Butterworth digital filter at a frequency range set between 20 and 500 Hz, and the maximal isometric force and the root mean square value of the sEMG signal were then determined using the highest 1-s epoch of the force–time signal.

### Functional testing

#### 30 s Sit-to-stand

Participants began the test seated on a chair (0.43 m height) with both arms crossed over their chest and feet shoulder-width apart and were instructed to stand up as many times as possible in 30 s. Only one attempt of this test was performed, and the number of repetitions was used for further analysis.

#### Gait Speed

Participants were instructed to walk a 10-m distance at their habitual and maximal gait speeds in separate trials. To avoid acceleration and deceleration interference, a 1-m distance both before and after the 10-m track was provided. The time required to cover the distance was measured using a stopwatch and the corresponding gait speed was determined in m∙s^−1^. Each participant was granted two attempts per speed with a 1-min interval between them and the best result was used for analysis.

#### Timed Up-and-go

The Timed Up-and-Go (TUG) test began with participants seated on a chair and a cone positioned 3 m in front of them. At the investigator’s command, participants rose from the chair, walked as fast as possible without running around the cone and returned to the initial seated position. The time required to perform the task was measured using a stopwatch and the lower of the values recorded in two attempts was used in the analysis.

#### Stair climbing

Participants were instructed to climb a flight of stairs (10 steps, 17 cm each) as fast as possible, without using the handrails. The time required to complete the task (i.e., the time from the moment the participants set their foot on the first step to when both feet were on the last step) was measured using a stopwatch. Each participant had two attempts interspersed by 1 min and the best time was used for analysis.

#### 6-min Walk Test

The test was performed on a 30-m flat surface in which participants were instructed to walk back and forth as many times as they could for 6 min. Encouragement was provided every minute (e.g., “You are doing well. There are X min left”). Participants performed the test once and the total distance covered was used in the analysis.

### Statistical analysis

Data are reported as mean ± standard deviation (SD) unless stated otherwise. Intraclass correlation coefficients (ICC) were calculated using the “*irr*” R package (Version 0.84.1). Two-way random effects models (ICC_2,1_) for absolute agreement without interaction effects were used for all outcomes, except for the ultrasound-derived measures which were determined using ICC_2,5_ models as both muscle thickness and echo-intensity values were originally calculated as the average of five measures [26]. Thresholds adopted to describe ICCs were the following: poor (< 0.50), moderate (0.50–0.75), good (0.75–0.90), and excellent (≥ 0.90) [27]. The standard error of measurement (SEM), as a measure of absolute error variation between the two measures (i.e., baseline and pre-training), was calculated as $SEM=\sqrt{{{\upsigma }^{2}}_{error}}$ [28]. The minimal detectable change for a 90% confidence interval (MDC_90_), considered as a threshold to differentiate between real and random changes for a certain test procedure, was further calculated as $MDC=\text{SEM}\times \sqrt{2}\times 1.65$. Finally, the within-individual coefficient of variation (CV) was determined using the root mean square approach described by Bland [29]. Specifically, the CV was calculated for each participant as the ratio of the variance and the squared mean of the two measurements, and then aggregated for the entire sample as the square root of the mean of this ratio as follows: $CV=\sqrt{\sum (\frac{{\sigma }_{i}^{2}}{{\overline{x} }_{i}^{2}})/n}$. Although no universally accepted thresholds for interpreting CV results are available, a CV value ≤ 10% is generally considered indicative of acceptable reliability. All statistical analyses were performed using R (v. 4.3.1; R Core Team, 2023), and the analysis code is available as a Supplementary File.

## Results

A total of 43 participants were successfully enrolled, and their characteristics are detailed in Table 1. While the majority of data were successfully collected, technical issues with data retrieval at the baseline time point affected a very small number of cases. Specifically, data could not be retrieved for two older adults for the MVIC and RF sEMG tests, as well as for two and one middle-aged adults on the LP and KE peak power tests at a single % 1-RM. To ensure consistency in comparisons across different % 1-RM in the peak power results, these participants were excluded from analyses involving other loads. The exact sample sizes for each analysis are clearly indicated in the relevant tables.

**Table 1:** Sample characteristics at baseline

	All sample (*n* = 43)	MID (*n* = 17)	OLD (*n* = 18)	LIM (*n* = 8)
	Mean ± SD	Mean ± SD	Mean ± SD	Mean ± SD
Age (years)	62.1 ± 12.8	48.8 ± 4.8*^†^	68.9 ± 6.5^†^	77.3 ± 8.2
Height (m)	1.62 ± 0.09	1.67 ± 0.09	1.66 ± 0.09	1.64 ± 0.07
Body mass (kg)	72.0 ± 13.2	73.3 ± 13.2	74.5 ± 14.7	70.2 ± 12.8
Body mass index	26.2 ± 3.2	26.0 ± 2.7	26.9 ± 4.3	25.9 ± 2.6
Waist circumference (cm)	89.8 ± 10.9	86.8 ± 10.1	93.7 ± 12.8	91.2 ± 9.6
SBP (mmHg)	123.6 ± 15.9	121.3 ± 13.9	126.0 ± 17.3	128.2 ± 20.1
DBP (mmHg)	71.7 ± 9.5	76.6 ± 9.5	71.4 ± 8.1	67.0 ± 8.8
SPPB (score)	11.0 ± 1.6	11.8 ± 0.5^†^	11.5 ± 0.7^†^	8.1 ± 1.0
Sex	21F & 22M	9F & 8M	9F & 9M	3F & 5M

*MID* Middle-aged adults; *OLD* Older adults; *LIM* Older adults with mobility limitations; *SBP* systolic blood pressure; *DBP* diastolic blood pressure; *SPPB* short physical performance battery score. * = significantly different from OLD (*p* < 0.05); † = significantly different from LIM (*p* < 0.05)

### Relative reliability

Test–retest reliability results for the entire sample are shown in Tables 2 and 3. Both dynamic and isometric strength measures displayed excellent reliability (ICCs = 0.96 to 0.99), whereas ultrasound MT and EI reliability results ranged from good to excellent (ICCs = 0.88 to 0.98). Functional performance measures also showed good to excellent reliability (ICCs = 0.78 to 0.92), whereas the ICCs for VL and RF sEMG were good (ICC = 0.86 and 0.85, respectively). Further, LP peak power results displayed good to excellent reliability (ICCs = 0.76 to 0.96), and KE reliability results were excellent throughout (ICCs = 0.91 to 0.98).

**Table 2:** Comparison between baseline (week −4) and pre-intervention (week 0) time points among the entire sample investigated

	Baseline	Pre	N	ICC (95% CI)	SEM	MDC_90_	CV (%)
Dynamic strength							
*LP 1‑RM (kg)*	147.3 ± 61.1	154.8 ± 60.6	43	0.960 (0.921, 0.979)	11.67	27.24	5.6
*KE 1‑RM (kg)*	37.9 ± 13.8	39.0 ± 15.1	43	0.986 (0.969, 0.993)	1.62	3.78	2.2
Isometric strength							
*MVIC (kgf)*	28.0 ± 12.0	28.5 ± 11.9	41	0.958 (0.923, 0.977)	2.45	5.72	7.0
*VL sEMG (µV)*	314.2 ± 219.4	316.1 ± 209.4	41	0.860 (0.753, 0.923)	80.95	187.25	14.3
*RF sEMG (µV)*	215.7 ± 141.6	241.4 ± 164.5	41	0.853 (0.735, 0.920)	57.10	137.32	19.8
Ultrasound measures							
*RF MT (mm)*	14.00 ± 3.59	13.89 ± 3.46	43	0.985 (0.972, 0.992)	0.61	1.42	4.6
*VI MT (mm)*	11.82 ± 3.72	11.7 ± 3.83	43	0.979 (0.961, 0.989)	0.77	1.79	6.6
*VL MT (mm)*	18.74 ± 3.65	18.61 ± 3.65	43	0.981 (0.964, 0.989)	0.72	1.67	4.3
*VM MT (mm)*	21.09 ± 5.44	21.74 ± 5.13	43	0.938 (0.885, 0.967)	1.77	4.13	8.7
*QUAD MT (mm)*	65.62 ± 14.56	65.94 ± 14.03	43	0.984 (0.971, 0.991)	2.55	5.95	3.9
*RF EI (a.u)*	116.85 ± 11.28	118.03 ± 10,75	43	0.920 (0.854, 0.957)	4.20	9.81	3.6
*VI EI (a.u)*	102.05 ± 12.46	103.83 ± 11.21	43	0.889 (0.796, 0.940)	5.22	12.17	5.2
*VL EI (a.u)*	106.07 ± 7.74	106.68 ± 7.74	43	0.879 (0.778, 0.935)	3.59	8.39	3.3
*VM EI (a.u)*	100.7 ± 10.69	103.66 ± 10.89	43	0.896 (0.768, 0.949)	4.33	10.10	4.6
*QUAD EI (a.u)*	106.42 ± 8.66	108.05 ± 8.20	43	0.895 (0.801, 0.944)	3.56	8.31	3.4
Functional capacity							
*30 s sit-to-stand (reps)*	14.9 ± 3.3	15.7 ± 4.2	43	0.817 (0.672, 0.900)	1.54	3.59	6.3
*Habitual gait speed (m*^*.*^*s*^*−1*^*)*	1.42 ± 0.24	1.41 ± 0.19	43	0.775 (0.621, 0.871)	0.10	0.24	5.5
*Maximal gait speed (m*^*.*^*s*^*−1*^*)*	2.00 ± 0.37	1.98 ± 0.42	43	0.805 (0.668, 0.890)	0.18	0.41	6.3
*Timed up-ang-go (s)*	6.55 ± 1.53	6.48 ± 1.63	43	0.917 (0.852, 0.954)	0.46	1.07	5.5
*Stair climb (s)*	4.84 ± 1.37	4.86 ± 1.34	43	0.893 (0.810, 0.940)	0.45	1.05	6.8
*6-min walk (m)*	575.8 ± 93.8	571.1 ± 100.0	43	0.905 (0.832, 0.947)	30.06	70.14	4.2

*LP* leg press; *KE* knee extension; *1‑RM* one repetition maximum; *MVIC* maximal voluntary isometric contraction; *VL* vastus lateralis; *RF* rectus femoris; *BF* biceps femoris; *sEMG* surface electromyography signal; *MT* muscle thickness; *QUAD* quadriceps; *EI* echo intensity value; *ICC* intraclass correlation coefficient; *95% CI* 95% confidence interval; *SEM* standard error of measurement; *MDC*_*90*_ minimal detectable change with 90% confidence

**Table 3:** Comparison between baseline (week −4) and pre-intervention (week 0) time points among the entire sample investigated

	Baseline	Pre	N	ICC (95% CI)	SEM	MDC	CV (%)
LP peak power							
*PP 30% (W)*	394.7 ± 279.5	409.7 ± 273.1	41	0.964 (0.934, 0.981)	52.10	121.58	10.3
*PP 40% (W)*	429.1 ± 271.9	443.9 ± 278.8	41	0.929 (0.872, 0.962)	73.31	171.06	10.8
*PP 50% (W)*	437.1 ± 256.5	462.5 ± 280.7	41	0.958 (0.919, 0.978)	52.84	123.30	9.4
*PP 60% (W)*	440.7 ± 252.5	470.6 ± 278.0	41	0.942 (0.888, 0.969)	61.45	143.39	9.8
*PP 70% (W)*	409.0 ± 229.6	445.5 ± 254.2	41	0.886 (0.790, 0.938)	79.06	184.48	12.8
*PP 80% (W)*	370.5 ± 200.5	405.0 ± 227.7	41	0.850 (0.733, 0.918)	80.74	188.40	14.4
*PP 90% (W)*	305.8 ± 160.0	359.5 ± 227.5	41	0.763 (0.572, 0.871)	90.56	211.33	18.3
KE peak power							
*PP 30% (W)*	368.3 ± 215.3	398.8 ± 244.8	42	0.920 (0.849, 0.957)	62.63	146.15	11.5
*PP 40% (W)*	378.3 ± 223.5	392.1 ± 247.6	42	0.969 (0.944, 0.984)	40.52	94.55	9.2
*PP 50% (W)*	395.8 ± 234.4	409.4 ± 248.7	42	0.979 (0.960, 0.989)	34.43	80.35	6.6
*PP 60% (W)*	398.2 ± 220.1	405.4 ± 237.0	42	0.972 (0.949, 0.985)	38.42	89.66	7.2
*PP 70% (W)*	380.4 ± 211.9	391.9 ± 215.2	42	0.960 (0.928, 0.978)	42.23	98.53	9.9
*PP 80% (W)*	343.4 ± 188.0	355.1 ± 198.7	42	0.949 (0.908, 0.972)	43.27	100.96	11.8
*PP 90% (W)*	311.4 ± 202.8	301.8 ± 183.4	42	0.910 (0.839, 0.950)	58.34	136.13	18.0

*LP* leg press; *KE* knee extension; *ICC* intraclass correlation coefficient; *95% CI* 95% confidence interval; *SEM* standard error of measurement; *MDC* minimal detectable change with 90% confidence

When the results were analyzed separately for each group, ICCs for dynamic and isometric strength measures were found to be excellent in all three groups investigated (ICCs = 0.91 to 0.99). Similar results were also observed for ultrasound MT outcomes (ICCs = 0.93 to 0.99), whereas middle-aged and healthy older adults tended to show better reliability results for some (e.g., RF and VI), but not all (VL, VM, and QUAD), EI outcomes as compared to mobility-limited older adults. Differences between the groups in the ICCs for the functional measures varied considerably between tests. As an example, 30-s sit-to-stand test ICCs were found to be moderate, good, and excellent in middle-aged, healthy older, and mobility-limited older adults (ICCs = 0.52, 0.78, and 0.93, respectively), whereas 6-min walk test results were classified as good in all three groups (ICCs = 0.78 to 0.79). Finally, middle-aged and healthy older adults tended to show better LP peak power reliability values between 30–70% 1‑RM (ICCs = 0.85 to 0.97 vs. 0.72 to 0.85), whereas the reproducibility of results at 80–90% 1‑RM was better in participants with mobility limitations (ICCs = 0.59 to 0.84 vs. 0.95 to 0.96, respectively). On the other hand, KE power measures ranged from good to excellent in all three groups (ICCs = 0.82 to 0.97), except for the 30 and 90% 1‑RM loads in those with mobility limitations, which were moderate (ICCs = 0.51 and 0.70, respectively). A full description of the test–retest reliability results for each of the three groups investigated is available as supplementary file (Supplementary Tables 1 and 2).

### Absolute reliability

The SEM, MDC and CV results for the entire sample are also available in Tables 2 and 3. Both dynamic and isometric strength measures displayed acceptable CV values (i.e., 2.2% to 7%). Likewise, ultrasound MT, EI and all functional performance measures demonstrated CV values under 10% (3.3% to 8.7%), whereas all sEMG measures exhibited high CV values (14.3% to 19.8%). Peak power, in turn, displayed acceptable CV values between 30–60% and 40–70% 1‑RM in the LP and KE exercises, respectively. The remaining loads analyzed showed slightly higher CV values (11.5% to 12.8%), except for the 80–90% 1‑RM trials in the LP (14.4% and 18.3%) and 90% in the KE (18%).

When results were analyzed separately for each group, CV values for dynamic and isometric strength measures were found acceptable in all three groups investigated (0.8% to 9%), despite values being somewhat larger in the mobility-limited participants for the LP 1‑RM and MVIC measures as compared to the other two groups (9% to 10.5% vs. 3.8% to 6.4%). Acceptable results were also observed for ultrasound MT (3.7% to 9.7%) and EI (2.6% to 5.7%), as well as for the functional performance measures (3.2% to 9.3%). sEMG measures, on the other hand, exhibited high CV values (13.2% to 23.5%) in all the groups, with the exception of the VL muscle in the middle-aged group (10.9%). Peak power, in turn, varied considerably between groups and exercises. For the middle-aged group, acceptable CV values were observed at 30% (10.3%) and 50% (8%) and 40–80% (4.3% to 9.2%) in the LP and KE exercise, respectively, whereas the CV for the remaining loads ranged from 12.5% to 20.1%. For the healthy older adults, CV values obtained for the LP exercise were acceptable from 30–60% 1‑RM (7.8% to 8.8%), but increased with rising intensity (10.5% at 70%, 14% at 80% and 20.7% at 90% 1‑RM). In the KE exercise, CV values indicated acceptable reliability between 50–70% 1‑RM (7.8% to 8.6%), but poorer reproducibility at the other loads tested (10.6% to 19.6%). Finally, participants with mobility limitations showed CV values under 10% at 50% and 60% 1‑RM (both 8.6%), slightly greater values at 30–40 and 80% 1‑RM (10.1% to 12.4%), and significantly poorer reliability at 70% and 90% (14% to 22.5%). A full description of the absolute reliability results for each of the three groups investigated is also available in Supplementary Tables 1 and 2.

## Discussion

The present study aimed at determining the test–retest reliability of commonly used neuromuscular, morphological, and functional test measures in a sample of middle-aged and older participants who presented with a wide range of functional abilities. Our main analysis suggests that most of these measures are sufficiently reliable, even when the two tests are performed a month apart from each other. Our study also supports the notion that, provided that the same equipment and procedures are used, the test–retest reliability of the measures is mostly comparable between the subsets of participants investigated.

The reliability of measures must always be evaluated in light of their intended application [30]. In studies examining the effects of aging or training interventions on muscle function, this necessitates ensuring that the anticipated changes associated with aging or the expected effects of the intervention significantly outweigh the fluctuations indicated by reliability statistics. In this regard, the typical errors reported in this paper indicate that the reliability of muscle function measures obtained from both the single and multi-joint exercises tested may be adequate for use in exercise intervention studies. As an example, peak power output improved by 24%−34% on average at loads between 30–90% 1‑RM in the LP after a 12-week high-velocity resistance training program in the present sample [22]. These improvements far outweigh the typical error values reported in the current manuscript, even at the 90% 1‑RM load.

**Fig. 1:**
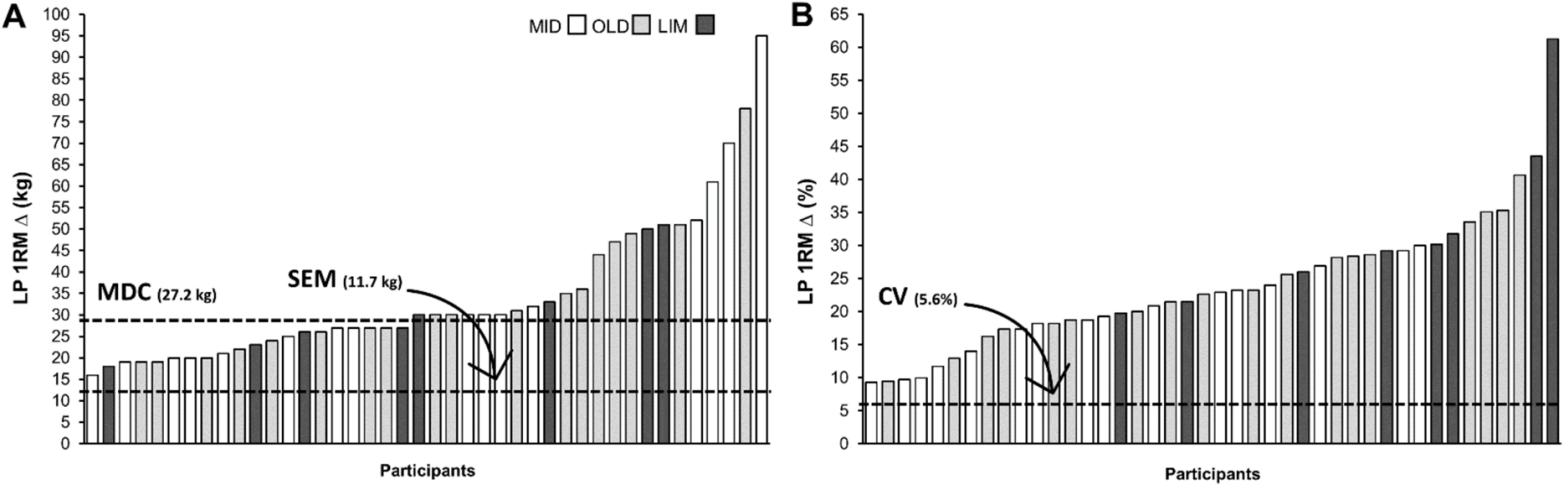
Leg press 1‑RM results (pre-post-training difference, ∆) from an exemplary 12-week high-velocity resistance training intervention (Schaun et al., 2022). The horizontal lines correspond to the standard error of measurement (SEM), minimal detectable change with 90% confidence (MDC), and the coefficient of variation (CV) as determined in the present study. LP = leg press; 1‑RM = one repetition maximum; MID = middle-aged adults; OLD = older adults; LIM = mobility-limited older adults

The present results also serve to expand on the findings of our main trial publication [22] by providing a background against which the individual results of the training intervention can be further scrutinized to confirm its efficacy. As previously emphasized by Perera et al. [31], it is crucial to consider whether estimates of measurement error or meaningful change, such as the SEM and the MDC, can be realistically achieved due to the investigated phenomenon (e.g., aging) or the manipulation of independent variables (e.g., exercise training). To further exemplify this, we included a figure (Fig. 1) displaying both the absolute and relative change in LP 1‑RM performance for each of the participants included in the aforementioned trial, as well as indicated the SEM, the MDC, and the CV values reported in Table 2 of the current manuscript.

When comparing the SEM results with the improvements of each participant in response to the 12-week high-velocity resistance training program, it becomes evident that every individual increased their LP 1‑RM performance by more than the SEM (11.7 kg). As a result, this finding gives us confidence that the differences observed on this measure were not attributable to measurement error, but rather reflect a systematic change in participants’ performance. Further comparison with the MDC (27.2 kg) provides an even more appropriate context for evaluating the efficacy of the intervention, given the sample variability. Specifically, our analysis suggests that approximately 52% of the entire sample experienced real changes in performance (i.e., not likely due to chance variation; [32]), with improvements observed for 22 out of 42 participants.

The previous example also enables us to compare the reliability results calculated for the entire sample (see Table 2) with those observed for each of the three groups (refer to Supplementary Table 1), and to assess its impact on the conclusions drawn from the intervention. As mentioned above, comparing the changes in LP 1‑RM performance against the MDC calculated for the entire sample suggests that approximately 50% of the sample experienced a real change in their maximal strength on the LP. A closer examination at Fig. 1 reveals that 8 of the 17 participants in the middle-aged group, 10 of the 18 participants in the healthy older adults’ group, and 4 of the 8 participants with mobility limitation exhibited a change in performance greater than the MDC. This frequency distribution, with roughly 50% of participants in each of the three groups, supports the notion that the capacity of older individuals to respond to training is maintained and comparable to that of younger participants, at least up to individuals with mild to moderate limitations in mobility.

Alternatively, applying the MDC values calculated for each individual group (39.2 kg, 11.8 kg, and 20.1 kg for the middle-aged, healthy older, and mobility-limited older adult participants, respectively) to the changes in performance observed for those specific participants presents a different perspective. This analysis suggests that approximately 71% of participants experienced a real change in their LP 1‑RM performance (data not shown), but with a notable shift in the frequency distribution within each group. While only 4 of the 17 middle-aged participants would be considered as having genuinely improved their 1‑RM value, all 26 participants in the two other groups would be considered to have experienced a real change in their maximal strength. As a consequence, these results would support a different conclusion than the one suggested above. Rather than highlighting the comparability of the older participants to those younger or possessing a better functional capacity, these findings would suggest that the training dose might not have been optimal for improving performance in the middle-aged participants or that a longer training period would be necessary for this specific population based on the training dose provided. Although caution is necessary in interpreting these results due to the relatively small sample sizes of the three individual groups, this real-life example underscores the importance of acquiring appropriate reliability estimates for the population one is interested in studying and their potential impact on conclusions driven from it.

This is also relevant in studies involving heterogeneous samples, where variations in functional capacity, health status, or mobility differences may be present. Significant variability in reliability between subsets of participants could introduce bias, confounding factors, or measurement errors, undermining the validity of pooled data and conclusions about intervention effects. In randomized clinical trials, reliable baseline and follow-up measurements are critical for detecting true intervention effects and distinguishing them from variability due to measurement error. Demonstrating consistent reliability across diverse subsets, such as middle-aged adults and older adults with mobility limitations, ensures these tests can be confidently applied, regardless of participant characteristics, thereby enhancing the confidence in and generalizability of findings. Conversely, substantial differences in reliability between these subsets could complicate comparisons both within and between study arms, particularly if subset representation is uneven or improperly accounted for in data analysis.

When considering the individual measures investigated, in the present study we demonstrate maximal strength measures derived from both dynamic and isometric tests to possess excellent reliability, both in the entire sample and when determined separately for each group. This is in line with previous research that showed similar results in middle-aged and older adults [33–35], including in those with mobility limitations [18, 19]. The fact that reliable results were obtained in both isometric and dynamic strength tests, as well as in single- and multi-joint exercises, suggests that researchers can select the appropriate maximal strength measure based on the specific muscle actions they are most interested in [35].

Maximal strength measures are often coupled with sEMG in an attempt to obtain additional information regarding the underlying organization and production of movement [36, 37]. In our investigation, sEMG measures showed good relative reliability, whereas absolute reliability was found to be less reproducible as compared to the other measures investigated. As suggested by Dutra et al. [38], the poorer agreement observed in the sEMG measures can be partly explained by the random nature of motor unit action potentials, which leads to greater variability in the sEMG signal as compared to that observed in measures such as muscle force. Consequently, the high intra-individual variability of the sEMG signals may limit the applicability of sEMG measures to assessing group mean responses to an intervention, at least in these particular muscles and with the procedures employed in the current study [39].

Muscle size and quality measures, on the other hand, exhibited excellent absolute test–retest reliability, whether assessed individually for each muscle or combined to represent the quadriceps femoris muscle. More importantly, the reliability outcomes remained comparable across the three groups, with consistently lower CV values observed for the RF and VL muscles, as well as for the aggregated quadriceps data. The results for the RF and VL muscles also fall within the range of those previously reported in older adults [40–44], even when compared to more valid measures of muscle size such as panoramic ultrasound-derived muscle cross-sectional area [42, 45]. Taken together, our findings reinforce the utility of ultrasound-derived MT and EI measures for comparative analyses of age- and health-related differences in muscle size and quality, as well as their responsiveness to interventions like physical exercise, even among individuals exhibiting significant reductions in muscle function.

As for the power measures, although CV values seemed slightly larger in the LP as compared to KE, results were mostly comparable between the two exercises. Moreover, when considering the different loads, results in the range of 30% to 70% 1‑RM typically showed good to excellent reliability and an acceptable CV. Peak power measures at higher loads, on the other hand, exhibited larger CV values, especially at 90% 1‑RM.

Possible explanations for these results could be related to the protocol used by us to assess peak power. Specifically, in the current study peak power was measured at each load using only a single repetition, whereas previous research in older adults with and without mobility limitations suggested that using two to three repetitions per load could be preferable for achieving more reliable results [19, 46]. In addition, the order of the loads during the power tests could not be randomized and, although unlikely [47], performance with the final loads might have been influenced by cumulative fatigue. Therefore, although the relative reliability results reported by us are within those reported for muscle power testing [47], our absolute reliability results could serve as a more conservative estimate of the reliability estimates.

Finally, functional measures also showed good to excellent reliability results. More importantly, the absolute reliability values were typically close to or below the clinically meaningful differences proposed for some of the tests investigated (e.g., [31, 48–50]). In addition, comparison between the groups suggests that differences in test–retest reliability do not follow a clear trend between the groups and should be considered separately for each test. As an example, while 30-s sit-to-stand performance showed lower CV in the group with mobility limitation, the 6-min walk test showed higher CV values in the same group, whereas results from the timed up-and-go test were similar between the three groups. It should be noted, however, that despite these differences in magnitude CV values were relatively low for all the groups.

### Considerations for future studies

Differences in test reliability can have significant implications for study design, particularly when comparing diverse groups or subsets of individuals. While the results of the current study indicate that reliability was relatively similar across groups for most measures investigated, it remains important to consider potential challenges and implications associated with reliability differences in future research.

Substantial variability in reliability between groups may complicate the interpretation of results. Low reliability within one group reduces the robustness of typically used parametric tests due to inhomogeneity of variance, which reduces statistical power. Similarly, high variance and uncertainty of true scores impact the interpretability of effect sizes. In the present study, reliability metrics, including SEM, MDC, and CV, were largely consistent across middle-aged participants, healthy older adults, and older adults with mobility limitations. However, subtle differences were observed for some measures, such as the 30-s sit-to-stand and 6-min walk tests.

To address these differences, future studies should prioritize improving measurement precision by means of enhancing the signal-to-noise ratio. Strategies to achieve this would include refining testing protocols, providing additional familiarization sessions, offering clearer explanations and cueing, repeating the test and averaging the scores to reduce noise, whenever possible, and using higher-resolution measurement tools. These steps can reduce variability and improve the reliability of test outcomes, thereby enabling more accurate comparisons across groups. A secondary approach, though less ideal, involves increasing sample sizes in groups with lower reliability. While this can mitigate some effects of variability in group-level comparisons, it is important to note that it does not resolve the underlying issue of individual-level measurement error.

Furthermore, researchers must recognize the limits of improving precision alone. Beyond a certain point, efforts to enhance measurement sensitivity may yield diminishing returns, particularly if the changes detected are not meaningful or clinically relevant. For example, detecting minuscule differences, such as nanoseconds in a TUG test, does not increase the signal-to-noise ratio and may not translate into practical or actionable insights. As such, intervention designs may also need to be planned in a way that increases the likelihood that the improvements observed will exceed the estimates of measurement error, such as planning interventions with a slightly longer duration or optimizing the intervention itself by carefully considering its components (e.g., intensity and volume, as in the case of exercise trials).

### Limitations

The present study is not without limitations. First, the small sample size for the group-specific analyses can potentially limit the external validity of our results and the precision of the estimates, especially for the participants with mobility limitations. Caution is also needed when extrapolating these results to severely limited individuals, as our sample included only individuals with mild to moderate limitations in mobility. Another possible limitation is the fact that sEMG amplitude data were not corrected to account for the influence of subcutaneous adipose tissue, even though no difference was previously found between original and corrected sEMG values when comparing middle-aged and older adults with and without mobility limitation (Clark et al., 2010). Finally, although the sex distribution was similar (~ 50%) in each of the subgroups included, the impact of sex on the reliability measures reported was not possible to be determined and should be taken into consideration.

Despite these limitations, the results of the present study offer valuable insights that can aid researchers in planning future trials. Specifically, the reported SEM, MDC, and CV values can guide researchers in estimating appropriate sample sizes for their studies, including the design of pilot investigations. Practitioners can also benefit from these findings by using them as a reference point to evaluate changes resulting from training or rehabilitation programs tailored to middle-aged and older individuals with functional profiles similar to those in this study. This approach can serve as a practical starting point until practitioners establish their own reliability measures, as is commonly recommended.

## Conclusion

Overall, our results suggest the majority of the physical and functional measures investigated to be reliable in middle-aged and older adults with varying functional capacities. Importantly, several tests exhibited consistent reliability results across the three participant subsets included, supporting the suitability of these measures for studies comparing these different populations.

## Supplementary Information

Below is the link to the electronic supplementary material.

Supplementary file1 (DOCX 62 KB)

Supplementary file2 (R 13 KB)

## Data Availability

Data from the present study will be made available upon request to the corresponding author.
