# Supplementary material for: Examining the test–retest reliability of commonly used neuromuscular, morphological, and functional measures in aging adults
Source: GeroScience. 2025 Mar 11;47(3):4381–93. doi: 10.1007/s11357-025-01590-0 (PMC12181469; doi:10.1007/s11357-025-01590-0)
Supplement: Supplementary file 1 — Supplementary file1 (DOCX 62 KB) [file 11357_2025_1590_MOESM1_ESM.docx]

*Examining the test-retest reliability of commonly used neuromuscular, morphological, and functional measures in aging adults*

GeroScience

Gustavo Z. Schaun^1,2*^, Peter Raidl^1^, Luana S. Andrade^2^, Gabriela B. David^2^, Eduardo F. Marins^2^, Mariana S. Häfele^2^, Stephanie S. Pinto^2^, Robert Csapo^1^, Cristine L. Alberton^2^

^1^Centre for Sport Science and University Sports, Department of Sport and Human Movement Science, University of Vienna, Vienna, Austria.

^2^Neuromuscular Assessment Laboratory, Physical Education School, Federal University of Pelotas, Pelotas, RS, Brazil.

*Corresponding author: gustavo.schaun@univie.ac.at

**Supplementary Table 1**. Comparison between baseline (wk -4) and pre-intervention (wk 0) time points among the groups investigated.

|  |  | **Baseline** |  | **Pre** |  | **N** |  | **ICC (95% CI)** |  | **SEM** |  | **MDC** |  | **CV (%)** |  |
| --- | --- | --- | --- | --- | --- | --- | --- | --- | --- | --- | --- | --- | --- | --- | --- |
| ***Dynamic strength*** |  |  |  |  |  |  |  |  |  |  |  |  |  |  |  |
| *LP 1RM (kg)* |  |  |  |  |  |  |  |  |  |  |  |  |  |  |  |
| Middle-aged adults |  | 183.7 ± 74.4 |  | 189.1 ± 68.9 |  | 17 |  | 0.944 (0.851-0.980) |  | 17.46 |  | 39.22 |  | 5.8 |  |
| Older adults |  | 139.2 ± 40.7 |  | 145.0 ± 40.0 |  | 18 |  | 0.975 (0.866-0.992) |  | 5.07 |  | 11.82 |  | 3.8 |  |
| Mobility-limited older adults |  | 95.6 ± 32.6 |  | 104.0 ± 35.2 |  | 8 |  | 0.911 (0.591-0.982) |  | 8.94 |  | 20.85 |  | 9.0 |  |
| *KE 1RM (kg)* |  |  |  |  |  |  |  |  |  |  |  |  |  |  |  |
| Middle-aged adults |  | 45.0 ± 16.4 |  | 46.2 ± 18.0 |  | 17 |  | 0.984 (0.955-0.994) |  | 2.02 |  | 5.08 |  | 2.1 |  |
| Older adults |  | 35.9 ± 10.9 |  | 37.1 ± 11.2 |  | 18 |  | 0.976 (0.921-0.991) |  | 1.54 |  | 3.60 |  | 2.9 |  |
| Mobility-limited older adults |  | 27.3 ± 7.4 |  | 27.5 ± 7.3 |  | 8 |  | 0.998 (0.989-0.999) |  | 0.33 |  | 0.76 |  | 0.8 |  |
|  |  |  |  |  |  |  |  |  |  |  |  |  |  |  |  |
| ***Isometric strength*** |  |  |  |  |  |  |  |  |  |  |  |  |  |  |  |
| *MVIC (kgf)* |  |  |  |  |  |  |  |  |  |  |  |  |  |  |  |
| Middle-aged adults |  | 33.6 ± 13.7 |  | 33.9 ± 13.0 |  | 17 |  | 0.962 (0.900-0.986) |  | 2.66 |  | 6.08 |  | 6.4 |  |
| Older adults |  | 26.1 ± 9.0 |  | 27.0 ± 9.8 |  | 16 |  | 0.928 (0.810-0.974) |  | 2.54 |  | 5.93 |  | 5.8 |  |
| Mobility-limited older adults |  | 20.0 ± 8.3 |  | 20.0 ± 6.5 |  | 8 |  | 0.937 (0.716-0.987) |  | 1.99 |  | 4.63 |  | 10.5 |  |
| *VL sEMG (µV)* |  |  |  |  |  |  |  |  |  |  |  |  |  |  |  |
| Middle-aged adults |  | 379.3 ± 273.6 |  | 395.4 ± 290.6 |  | 17 |  | 0.950 (0.870-0.982) |  | 63.88 |  | 149.07 |  | 10.9 |  |
| Older adults |  | 255.4 ± 98.4 |  | 284.8 ± 102.5 |  | 18 |  | 0.831 (0.544-0.940) |  | 37.64 |  | 87.84 |  | 13.2 |  |
| Mobility-limited older adults |  | 293.3 ± 254.2 |  | 210.0 ± 67.9 |  | 8 |  | 0.415 (-0.274-0.842) |  | 140.81 |  | 328.58 |  | 23.5 |  |
| *RF sEMG (µV)* |  |  |  |  |  |  |  |  |  |  |  |  |  |  |  |
| Middle-aged adults |  | 259.1 ± 170.7 |  | 290.6 ± 231.0 |  | 17 |  | 0.909 (0.768-0.966) |  | 59.06 |  | 137.82 |  | 19.0 |  |
| Older adults |  | 172.3 ± 83.8 |  | 219.4 ± 82.0 |  | 16 |  | 0.633 (0.125-0.864) |  | 43.56 |  | 101.65 |  | 21.3 |  |
| Mobility-limited older adults |  | 210.1 ± 154.9 |  | 180.9 ± 84.4 |  | 8 |  | 0.729 (0.156-0.938) |  | 65.65 |  | 153.18 |  | 18.4 |  |
|  |  |  |  |  |  |  |  |  |  |  |  |  |  |  |  |
| ***Ultrasound measures*** |  |  |  |  |  |  |  |  |  |  |  |  |  |  |  |
| *RF MT (mm)* |  |  |  |  |  |  |  |  |  |  |  |  |  |  |  |
| Middle-aged adults |  | 15.4 ± 2.9 |  | 15.2 ± 2.9 |  | 17 |  | 0.972 (0.925-0.990) |  | 0.68 |  | 1.58 |  | 4.4 |  |
| Older adults |  | 13.4 ± 4.1 |  | 13.4 ± 3.8 |  | 18 |  | 0.993 (0.980-997) |  | 0.49 |  | 1.14 |  | 4.4 |  |
| Mobility-limited older adults |  | 12.4 ± 3.0 |  | 12.2 ± 3.1 |  | 8 |  | 0.972 (0.864-0.994) |  | 0.75 |  | 1.74 |  | 5.7 |  |
| *VI MT (mm)* |  |  |  |  |  |  |  |  |  |  |  |  |  |  |  |
| Middle-aged adults |  | 14.1 ± 3.4 |  | 13.9 ± 3.9 |  | 17 |  | 0.969 (0.917-0.989) |  | 0.90 |  | 2.11 |  | 6.1 |  |
| Older adults |  | 11.4 ± 3.1 |  | 11.4 ± 2.9 |  | 18 |  | 0.969 (0.916-0.988) |  | 0.75 |  | 1.75 |  | 6.9 |  |
| Mobility-limited older adults |  | 8.1 ± 2.2 |  | 7.8 ± 2.2 |  | 8 |  | 0.972 (0.870-0.994) |  | 0.50 |  | 1.16 |  | 6.8 |  |
| *VL MT (mm)* |  |  |  |  |  |  |  |  |  |  |  |  |  |  |  |
| Middle-aged adults |  | 20.9 ± 1.9 |  | 20.7 ± 2.2 |  | 17 |  | 0.933 (0.820-0.976) |  | 0.74 |  | 1.72 |  | 3.8 |  |
| Older adults |  | 17.9 ± 3.8 |  | 18.0 ± 3.9 |  | 18 |  | 0.984 (0.957-0.994) |  | 0.70 |  | 1.63 |  | 4.4 |  |
| Mobility-limited older adults |  | 16.0 ± 4.0 |  | 15.7 ± 3.4 |  | 8 |  | 0.980 (0.908-0.996) |  | 0.73 |  | 1.71 |  | 5.0 |  |
| *VM MT (mm)* |  |  |  |  |  |  |  |  |  |  |  |  |  |  |  |
| Middle-aged adults |  | 24.1 ± 4.7 |  | 23.9 ± 5.1 |  | 17 |  | 0.916 (0.768-0.970) |  | 1.97 |  | 4.59 |  | 8.1 |  |
| Older adults |  | 19.6 ± 5.1 |  | 21.2 ± 4.8 |  | 18 |  | 0.926 (0.681-0.976) |  | 1.55 |  | 3.61 |  | 9.7 |  |
| Mobility-limited older adults |  | 18.2 ± 5.2 |  | 18.5 ± 4.5 |  | 8 |  | 0.959 (0.798-0.992) |  | 1.42 |  | 3.32 |  | 7.2 |  |
| *QUAD MT (mm)* |  |  |  |  |  |  |  |  |  |  |  |  |  |  |  |
| Middle-aged adults |  | 74.4 ± 11.2 |  | 73.6 ± 11.9 |  | 17 |  | 0.967 (0.912-0.988) |  | 2.93 |  | 6.83 |  | 3.7 |  |
| Older adults |  | 62.2 ± 14.0 |  | 64.0 ± 13.2 |  | 18 |  | 0.984 (0.941-0.995) |  | 2.07 |  | 4.82 |  | 4.0 |  |
| Mobility-limited older adults |  | 54.6 ± 12.1 |  | 54.1 ± 10.9 |  | 8 |  | 0.986 (0.935-0.997) |  | 1.98 |  | 4.61 |  | 3.7 |  |
| *RF EI (a.u)* |  |  |  |  |  |  |  |  |  |  |  |  |  |  |  |
| Middle-aged adults |  | 110.6 ± 10.4 |  | 114.1 ± 10.4 |  | 17 |  | 0.906 (0.646-0.969) |  | 3.75 |  | 8.75 |  | 3.9 |  |
| Older adults |  | 118.5 ± 8.8 |  | 119.7 ± 9.8 |  | 18 |  | 0.940 (0.843-0.977) |  | 3.10 |  | 7.24 |  | 2.6 |  |
| Mobility-limited older adults |  | 126.6 ± 10.8 |  | 122.8 ± 12.2 |  | 8 |  | 0.862 (0.383-0.972) |  | 5.44 |  | 12.69 |  | 4.7 |  |
| *VI EI (a.u)* |  |  |  |  |  |  |  |  |  |  |  |  |  |  |  |
| Middle-aged adults |  | 95.5 ± 11.9 |  | 99.1 ± 12.2 |  | 17 |  | 0.882 (0.654-0.958) |  | 5.18 |  | 12.08 |  | 5.7 |  |
| Older adults |  | 103.2 ± 11.3 |  | 105.2 ± 9.0 |  | 18 |  | 0.888 (0.707-0.958) |  | 4.49 |  | 10.47 |  | 4.4 |  |
| Mobility-limited older adults |  | 113.3 ± 7.11 |  | 110.8 ± 10.2 |  | 8 |  | 0.678 (-0.577-0.935) |  | 6.22 |  | 14.51 |  | 5.8 |  |
| *VL EI (a.u)* |  |  |  |  |  |  |  |  |  |  |  |  |  |  |  |
| Middle-aged adults |  | 102.7 ± 6.6 |  | 103.2 ± 7.3 |  | 17 |  | 0.801 (0.442-0.928) |  | 4.08 |  | 9.52 |  | 3.7 |  |
| Older adults |  | 106.8 ± 7.1 |  | 108.0 ± 7.5 |  | 18 |  | 0.865 (0.647-0.949) |  | 3.55 |  | 8.28 |  | 3.2 |  |
| Mobility-limited older adults |  | 111.8 ± 8.5 |  | 111.1 ± 6.4 |  | 8 |  | 0.935 (0.680-0.987) |  | 2.74 |  | 6.40 |  | 2.3 |  |
| *VM EI (a.u)* |  |  |  |  |  |  |  |  |  |  |  |  |  |  |  |
| Middle-aged adults |  | 94.1 ± 6.5 |  | 97.9 ± 9.7 |  | 17 |  | 0.759 (0.303-0.914) |  | 4.80 |  | 11.20 |  | 5.2 |  |
| Older adults |  | 100.8 ± 9.2 |  | 104.8 ± 9.7 |  | 18 |  | 0.887 (0.484-0.965) |  | 3.52 |  | 8.21 |  | 4.3 |  |
| Mobility-limited older adults |  | 114.6 ± 7.3 |  | 113.6 ± 8.7 |  | 8 |  | 0.844 (0.196-0.969) |  | 4.33 |  | 10.09 |  | 3.5 |  |
| *QUAD EI (a.u)* |  |  |  |  |  |  |  |  |  |  |  |  |  |  |  |
| Middle-aged adults |  | 100.7 ± 7.0 |  | 103.5 ± 7.9 |  | 17 |  | 0.814 (0.461-0.934) |  | 3.91 |  | 9.13 |  | 4.1 |  |
| Older adults |  | 107.3 ± 6.7 |  | 109.4 ± 7.0 |  | 18 |  | 0.891 (0.673-0.961) |  | 2.80 |  | 6.53 |  | 2.9 |  |
| Mobility-limited older adults |  | 116.5 ± 5.6 |  | 114.5 ± 6.5 |  | 8 |  | 0.788 (0.062-0.957) |  | 3.54 |  | 8.27 |  | 3.1 |  |
|  |  |  |  |  |  |  |  |  |  |  |  |  |  |  |  |
| ***Functional capacity*** |  |  |  |  |  |  |  |  |  |  |  |  |  |  |  |
| *30STS (reps)* |  |  |  |  |  |  |  |  |  |  |  |  |  |  |  |
| Middle-aged adults |  | 17.3 ± 2.3 |  | 18.7 ± 3.5 |  | 17 |  | 0.516 (0.092-0.789) |  | 1.95 |  | 4.55 |  | 7.5 |  |
| Older adults |  | 14.4 ± 2.5 |  | 14.8 ± 3.3 |  | 18 |  | 0.775 (0.500-0.909) |  | 1.40 |  | 3.26 |  | 6.3 |  |
| Mobility-limited older adults |  | 10.6 ± 1.9 |  | 11.3 ± 2.1 |  | 8 |  | 0.927 (0.279-0.988) |  | 0.37 |  | 0.85 |  | 3.9 |  |
| *HGS (m^.^s^-1^)* |  |  |  |  |  |  |  |  |  |  |  |  |  |  |  |
| Middle-aged adults |  | 1.57 ± 0.24 |  | 1.54 ± 0.16 |  | 17 |  | 0.701 (0.351-0.880) |  | 0.11 |  | 0.26 |  | 5.3 |  |
| Older adults |  | 1.37 ± 0.19 |  | 1.36 ± 0.16 |  | 18 |  | 0.588 (0.172-0.824) |  | 0.11 |  | 0.27 |  | 6.7 |  |
| Mobility-limited older adults |  | 1.23 ± 0.14 |  | 1.23 ± 0.14 |  | 8 |  | 0.847 (0.395-0.968) |  | 0.06 |  | 0.13 |  | 3.2 |  |
| *MGS (m^.^s^-1^)* |  |  |  |  |  |  |  |  |  |  |  |  |  |  |  |
| Middle-aged adults |  | 2.24 ± 0.28 |  | 2.19 ± 0.38 |  | 17 |  | 0.658 (0.274-0.861) |  | 0.20 |  | 0.46 |  | 5.7 |  |
| Older adults |  | 1.94 ± 0.31 |  | 1.97 ± 0.40 |  | 18 |  | 0.813 (0.568-0.926) |  | 0.16 |  | 0.37 |  | 5.6 |  |
| Mobility-limited older adults |  | 1.62 ± 0.28 |  | 1.56 ± 0.21 |  | 8 |  | 0.504 (-0.275-0.878) |  | 0.18 |  | 0.42 |  | 9.3 |  |
| *TUG (s)* |  |  |  |  |  |  |  |  |  |  |  |  |  |  |  |
| Middle-aged adults |  | 5.50 ± 0.73 |  | 5.42 ± 0.63 |  | 17 |  | 0.720 (0.379-0.889) |  | 0.37 |  | 0.86 |  | 5.6 |  |
| Older adults |  | 6.55 ± 0.76 |  | 6.37 ± 0.85 |  | 18 |  | 0.759 (0.473-0.902) |  | 0.39 |  | 0.90 |  | 5.0 |  |
| Mobility-limited older adults |  | 8.79 ± 1.75 |  | 8.94 ± 1.92 |  | 8 |  | 0.852 (0.427-0.968) |  | 0.74 |  | 1.73 |  | 6.0 |  |
| *SC (s)* |  |  |  |  |  |  |  |  |  |  |  |  |  |  |  |
| Middle-aged adults |  | 4.01 ± 0.55 |  | 4.12 ± 0.45 |  | 17 |  | 0.614 (0.217-0.839) |  | 0.31 |  | 0.73 |  | 5.8 |  |
| Older adults |  | 4.71 ± 0.65 |  | 4.58 ± 0.49 |  | 18 |  | 0.469 (0.025-0.761) |  | 0.42 |  | 0.98 |  | 7.0 |  |
| Mobility-limited older adults |  | 6.89 ± 1.73 |  | 7.04 ± 1.66 |  | 8 |  | 0.839 (0.390-0.966) |  | 0.71 |  | 1.66 |  | 8.5 |  |
| *6MW (m)* |  |  |  |  |  |  |  |  |  |  |  |  |  |  |  |
| Middle-aged adults |  | 656.2 ± 61.8 |  | 651.3 ± 67.6 |  | 17 |  | 0.784 (0.498-0.916) |  | 30.66 |  | 71.54 |  | 3.3 |  |
| Older adults |  | 547.1 ± 56.7 |  | 553.0 ± 58.3 |  | 18 |  | 0.777 (0.500-0.910) |  | 27.50 |  | 64.17 |  | 4.0 |  |
| Mobility-limited older adults |  | 469.9 ± 75.4 |  | 441.2 ± 74.9 |  | 8 |  | 0.789 (0.270-0.945) |  | 31.05 |  | 72.45 |  | 6.7 |  |

LP = leg press; KE = knee extension; 1RM = one repetition maximum; MVIC = maximal voluntary isometric contraction; VL = vastus lateralis; RF = rectus femoris; BF = biceps femoris; sEMG = surface electromyography signal; MT = muscle thickness; QUAD = quadriceps; EI = echo intensity value; 30STS = 30s sit-to-stand; HGS = habitual gait speed; MGS = maximal gait speed; TUG = timed up-and-go; SC = stair climb; 6MW = 6-min walk; ICC = intraclass correlation coefficient; 95% CI = 95% confidence interval; SEM = standard error of measurement; MDC = minimal detectable change with 90% confidence.

**Supplementary Table 2**. Comparison between baseline (wk -4) and pre-intervention (wk 0) time points among the groups investigated.

|  |  | **Baseline** |  | **Pre** |  | **N** |  | **ICC (95% CI)** |  | **SEM** |  | **MDC** |  | **CV (%)** |  |
| --- | --- | --- | --- | --- | --- | --- | --- | --- | --- | --- | --- | --- | --- | --- | --- |
| ***LP peak power*** |  |  |  |  |  |  |  |  |  |  |  |  |  |  |  |
| *PP 30% (W)* |  |  |  |  |  |  |  |  |  |  |  |  |  |  |  |
| Middle-aged adults |  | 564.3 ± 331.5 |  | 582.7 ± 325.9 |  | 15 |  | 0.949 (0.857-0.982) |  | 75.81 |  | 176.90 |  | 10.3 |  |
| Older adults |  | 355.9 ± 198.4 |  | 364.9 ± 181.9 |  | 18 |  | 0.965 (0.910-0.987) |  | 36.19 |  | 84.44 |  | 8.8 |  |
| Mobility-limited older adults |  | 164.0 ± 69.12 |  | 186.2 ± 84.2 |  | 8 |  | 0.852 (0.447-0.968) |  | 27.37 |  | 63.87 |  | 13.9 |  |
| *PP 40% (W)* |  |  |  |  |  |  |  |  |  |  |  |  |  |  |  |
| Middle-aged adults |  | 594.4 ± 316.8 |  | 603.4 ± 332.7 |  | 15 |  | 0.889 (0.700-0.961) |  | 111.63 |  | 260.48 |  | 13.4 |  |
| Older adults |  | 398.0 ± 191.0 |  | 410.4 ± 196.1 |  | 18 |  | 0.969 (0.922-0.988) |  | 33.62 |  | 78.45 |  | 7.8 |  |
| Mobility-limited older adults |  | 189.0 ± 78.4 |  | 220.3 ± 121.7 |  | 8 |  | 0.719 (0.162-0.935) |  | 53.30 |  | 124.36 |  | 12.8 |  |
| *PP 50% (W)* |  |  |  |  |  |  |  |  |  |  |  |  |  |  |  |
| Middle-aged adults |  | 605.1 ± 290.1 |  | 625.1 ± 332.0 |  | 15 |  | 0.959 (0.885-0.986) |  | 63.90 |  | 149.12 |  | 8 |  |
| Older adults |  | 403.0 ± 170.2 |  | 425.1 ± 195.2 |  | 18 |  | 0.946 (0.860-0.980) |  | 40.85 |  | 95.32 |  | 7.9 |  |
| Mobility-limited older adults |  | 198.6 ± 93.6 |  | 242.0 ± 146.9 |  | 8 |  | 0.744 (0.215-0.941) |  | 59.21 |  | 138.16 |  | 15.4 |  |
| *PP 60% (W)* |  |  |  |  |  |  |  |  |  |  |  |  |  |  |  |
| Middle-aged adults |  | 595.6 ± 306.8 |  | 635.9 ± 322.9 |  | 15 |  | 0.931 (0.811-0.976) |  | 80.58 |  | 188.02 |  | 12.5 |  |
| Older adults |  | 404.8 ± 159.2 |  | 430.8 ± 192.9 |  | 18 |  | 0.943 (0.843-0.979) |  | 39.31 |  | 91.72 |  | 7.8 |  |
| Mobility-limited older adults |  | 231.0 ± 98.0 |  | 250.3 ± 156.2 |  | 8 |  | 0.745 (0.153-0.943) |  | 68.12 |  | 158.95 |  | 9.1 |  |
| *PP 70% (W)* |  |  |  |  |  |  |  |  |  |  |  |  |  |  |  |
| Middle-aged adults |  | 533.8 ± 288.9 |  | 582.4 ± 302.7 |  | 15 |  | 0.863 (0.650-0.951) |  | 107.80 |  | 251.55 |  | 15.5 |  |
| Older adults |  | 394.0 ± 140.9 |  | 418.9 ± 185.4 |  | 18 |  | 0.852 (0.654-0.941) |  | 62.77 |  | 146.47 |  | 10.5 |  |
| Mobility-limited older adults |  | 208.6 ± 78.9 |  | 248.6 ± 127.1 |  | 8 |  | 0.739 (0.199-0.940) |  | 50.68 |  | 118.27 |  | 13.0 |  |
| *PP 80% (W)* |  |  |  |  |  |  |  |  |  |  |  |  |  |  |  |
| Middle-aged adults |  | 477.2 ± 255.0 |  | 533.0 ± 267.3 |  | 15 |  | 0.844 (0.601-0.944) |  | 99.58 |  | 232.36 |  | 18.2 |  |
| Older adults |  | 345.7 ± 122.3 |  | 381.2 ± 166.5 |  | 18 |  | 0.693 (0.360-0.871) |  | 79.80 |  | 186.21 |  | 14.0 |  |
| Mobility-limited older adults |  | 226.4 ± 116.3 |  | 218.6 ± 98.7 |  | 8 |  | 0.964 (0.844-0.993) |  | 21.03 |  | 49.07 |  | 8.2 |  |
| *PP 90% (W)* |  |  |  |  |  |  |  |  |  |  |  |  |  |  |  |
| Middle-aged adults |  | 385.0 ± 204.7 |  | 487.1 ± 291.6 |  | 15 |  | 0.738 (0.328-0.907) |  | 115.91 |  | 270.47 |  | 20.1 |  |
| Older adults |  | 284.9 ± 103.2 |  | 327.6 ± 141.4 |  | 18 |  | 0.589 (0.202-0.821) |  | 77.15 |  | 180.03 |  | 20.7 |  |
| Mobility-limited older adults |  | 204.1 ± 100.9 |  | 192.3 ± 88.0 |  | 8 |  | 0.951 (0.794-0.990) |  | 20.66 |  | 48.21 |  | 9.4 |  |
|  |  |  |  |  |  |  |  |  |  |  |  |  |  |  |  |
| ***KE peak power*** |  |  |  |  |  |  |  |  |  |  |  |  |  |  |  |
| *PP 30% (W)* |  |  |  |  |  |  |  |  |  |  |  |  |  |  |  |
| Middle-aged adults |  | 518.1± 245.1 |  | 559.0 ± 301.1 |  | 16 |  | 0.908 (0.761-0.967) |  | 80.84 |  | 188.65 |  | 11.5 |  |
| Older adults |  | 314.3 ± 138.2 |  | 338.1 ± 134.1 |  | 18 |  | 0.839 (0.627-0.936) |  | 53.69 |  | 125.28 |  | 12.2 |  |
| Mobility-limited older adults |  | 190.3 ± 35.3 |  | 214.9 ± 78.4 |  | 8 |  | 0.509 (-0.168-0.999) |  | 42.09 |  | 98.22 |  | 10.1 |  |
| *PP 40% (W)* |  |  |  |  |  |  |  |  |  |  |  |  |  |  |  |
| Middle-aged adults |  | 520.2 ± 271.6 |  | 535.9 ± 314.2 |  | 16 |  | 0.970 (0.917-0.989) |  | 51.57 |  | 120.35 |  | 7.1 |  |
| Older adults |  | 321.3 ± 140.7 |  | 339.1 ± 148.3 |  | 18 |  | 0.941 (0.850-0.978) |  | 33.69 |  | 78.61 |  | 10.6 |  |
| Mobility-limited older adults |  | 222.4 ± 64.4 |  | 223.9 ± 85.0 |  | 8 |  | 0.834 (0.356-0.965) |  | 32.43 |  | 75.68 |  | 10.4 |  |
| *PP 50% (W)* |  |  |  |  |  |  |  |  |  |  |  |  |  |  |  |
| Middle-aged adults |  | 543.8 ± 275.2 |  | 550.7 ± 313.9 |  | 16 |  | 0.974 (0.927-0.991) |  | 49.05 |  | 114.46 |  | 4.3 |  |
| Older adults |  | 348.0 ± 156.9 |  | 368.4 ± 148.8 |  | 18 |  | 0.969 (0.891-0.989) |  | 23.58 |  | 55.02 |  | 7.8 |  |
| Mobility-limited older adults |  | 207.3 ± 57.1 |  | 218.7 ± 77.0 |  | 8 |  | 0.924 (0.695-0.984) |  | 18.05 |  | 42.12 |  | 8.6 |  |
| *PP 60% (W)* |  |  |  |  |  |  |  |  |  |  |  |  |  |  |  |
| Middle-aged adults |  | 546.5 ± 243.8 |  | 551.9 ± 277.7 |  | 16 |  | 0.960 (0.890-0.986) |  | 53.62 |  | 125.13 |  | 5.9 |  |
| Older adults |  | 345.4 ± 153.6 |  | 355.8 ± 161.1 |  | 18 |  | 0.967 (0.917-0.988) |  | 28.26 |  | 65.94 |  | 7.8 |  |
| Mobility-limited older adults |  | 220.6 ± 75.1 |  | 223.9 ± 86.9 |  | 8 |  | 0.930 (0.691-0.985) |  | 22.80 |  | 53.20 |  | 8.6 |  |
| *PP 70% (W)* |  |  |  |  |  |  |  |  |  |  |  |  |  |  |  |
| Middle-aged adults |  | 516.0 ± 252.3 |  | 523.1 ± 260.7 |  | 16 |  | 0.971 (0.919-0.990) |  | 45.08 |  | 105.20 |  | 9.2 |  |
| Older adults |  | 330.1 ± 132.3 |  | 345.0 ± 133.9 |  | 18 |  | 0.881 (0.717-0.954) |  | 45.89 |  | 107.08 |  | 8.6 |  |
| Mobility-limited older adults |  | 222.3 ± 83.6 |  | 234.9 ± 95.0 |  | 8 |  | 0.887 (0.567-0.976) |  | 30.62 |  | 71.45 |  | 14.0 |  |
| *PP 80% (W)* |  |  |  |  |  |  |  |  |  |  |  |  |  |  |  |
| Middle-aged adults |  | 453.9 ± 222.9 |  | 477.0 ± 238.9 |  | 16 |  | 0.947 (0.859-0.981) |  | 52.18 |  | 121.77 |  | 8.2 |  |
| Older adults |  | 299.7 ± 138.8 |  | 309.7 ± 126.1 |  | 18 |  | 0.911 (0.783-0.966) |  | 39.90 |  | 93.11 |  | 14.7 |  |
| Mobility-limited older adults |  | 220.7 ± 60.6 |  | 213.8 ± 91.2 |  | 8 |  | 0.860 (0.457-0.970) |  | 30.24 |  | 70.57 |  | 12.4 |  |
| *PP 90% (W)* |  |  |  |  |  |  |  |  |  |  |  |  |  |  |  |
| Middle-aged adults |  | 408.2 ± 237.9 |  | 401.4 ± 227.8 |  | 16 |  | 0.933 (0.821-0.976) |  | 61.72 |  | 144.02 |  | 14.0 |  |
| Older adults |  | 280.6 ± 174.4 |  | 274.0 ± 119.7 |  | 18 |  | 0.818 (0.576-0.928) |  | 65.17 |  | 152.07 |  | 19.6 |  |
| Mobility-limited older adults |  | 187.0 ± 65.2 |  | 165.2 ± 71.3 |  | 8 |  | 0.700 (0.125-0.930) |  | 36.77 |  | 85.79 |  | 22.5 |  |

LP = leg press; KE = knee extension; PP = peak power; ICC = intraclass correlation coefficient; 95% CI = 95% confidence interval; SEM = standard error of measurement; MDC_90_ = minimal detectable change with 90% confidence.
